# The association between oral health and nutritional status in older adults: a cross-sectional study

**DOI:** 10.1186/s12877-022-03133-0

**Published:** 2022-06-10

**Authors:** Claudine Khoury, Johan Samot, Catherine Helmer, Rafael Weber Rosa, Aurore Georget, Jean-François Dartigues, Elise Arrivé

**Affiliations:** 1 Université de Bordeaux, UFR d’Odontologie, Bordeaux, France; 2 CHU de Bordeaux, Pôle de médecine et chirurgie bucco-dentaire, Bordeaux, France; 3 Université de Bordeaux, INSERM, Bordeaux Population Health Research Center, UMR 1219, Bordeaux, France; 4 Université de Bordeaux, ISPED, Bordeaux, France; 5 CHU de Bordeaux, USMR, Pôle de Santé publique, Bordeaux, France

**Keywords:** Malnutrition, Oral health, Aged, Epidemiology, Mini Nutritional Assessment, Xerostomia, Dental occlusion

## Abstract

**Objectives:**

This work aimed to describe the nutritional status of French older adults (age ≥ 90 years) and studied the association between oral health and nutritional status.

**Methods:**

A cross-sectional study was carried out in 2014 among the participants of a cohort on cerebral and functional aging in France at their 25-year follow up (the PAQUID cohort). Nutritional status (Mini Nutritional Assessment [MNA]) and oral health status (number of decayed, missing, and filled teeth [DMFT], number of posterior occluding pairs, xerostomia [Xerostomia Inventory], and prosthetic rehabilitation) were recorded at the participants’ living places by two dentists. Univariate and multivariate logistic regressions were used to explore the association between oral health and nutritional status, with adjustments for potential confounders. Odds ratios (OR) were estimated with their 95% confidence interval (CI).

**Results:**

87 participants were included in the analyses: 74.7% were females and the mean age was 94.1 years (± 3.0). Malnutrition or risk of malnutrition (MNA < 24) was present in 23 participants (26.4%), with only one having malnutrition. The mean DMFT score was 26.5 (± 5.3). The mean number of posterior occluding pairs was 1.5 (± 2.3). Twenty-one participants had xerostomia (24.1%). Only 8.1% of the participants had all their teeth or adequate dentures; 47.1% had inadequate dentures, while 44.8% had no dentures despite tooth loss. After adjustment, xerostomia (OR = 8.79; 95% CI = 2.38–39.10; *p* = 0.002) was found to be associated with malnutrition or risk of malnutrition.

**Conclusion:**

Being at risk of malnutrition was common among people ≥ 90 years old and was associated with xerostomia. NCT04065828.

## Introduction

Nutritional status is an important factor for general health in older adults [1]. Studies have reported a prevalence of undernourishment in older adults, between 4.3% and 90%, because of differences in the study populations and definitions [2]. The underlying causes can be divided into primary (cognitive, behavioural, taste, and olfactory disorders) and secondary factors (adverse drug effects and comorbidities) [3]. These factors can lead to a loss of appetite, refusal to eat, and inability to prepare meals [3]. Malnutrition can increase the risk of falls [4] and frailty [5], and can lead to depression, decreased functional capacity, increased rates of infection, longer hospital stays, increased mortality, and increased economic burdens on the patient and society [2]. A French study showed that 7.4–14.8% of people aged 65 years and above were undernourished based on the Mini Nutritional Assessment (MNA), and the associated factors were age, widowhood, low body mass index (BMI), dementia, depression, dependency, use of more than three drugs per day, low level of education, and low income [6].

Oral problems are common in older adults. Comorbidities and their treatments, as well as dependence, can cause or accentuate oral pathologies and complicate their management [7]. Therefore, older individuals are more likely to have certain oral conditions such as chronic periodontitis, root caries, precancerous and cancerous conditions, oral infections, xerostomia, also known as dry mouth, loss of teeth, and denture use [8–11]. Oral problems are likely to contribute to the altered nutritional status in older adults via avoidance of certain foods because of masticatory problems or oral pain. This may lead to digestive disorders [12]. In addition, oral health problems can affect the quality of life, social relationships, and diet in this population [2].

However, the relationship between oral status and nutritional status in older adults is still neglected. Most studies have been conducted in specific populations only, such as institutionalized individuals [2], and only four studies conducted in the general geriatric population could be identified in a recent systematic review including ten studies [13]. Associations were found between hard and soft tissue conditions of the mouth, xerostomia and subjective oral health and poor nutritional status. A meta-analysis showed that well-nourished subjects had a higher number of pairs of teeth compared to individuals with poor nutritional status who had a lower mean number of present teeth [14]. Furthermore, there were no studies that addressed people aged 90 years and above. The aim of this study was to investigate the nutritional status of French older adults (age ≥ 90 years) and the association between oral health status and nutritional status in this population. We hypothesized that poor oral health was associated with impaired nutritional status.

## Methods

### Study population and design

A cross-sectional oral sub-study was conducted on consenting participants who were seen at the 25-year follow up of the Personnes Agées QUID (PAQUID) cohort study [15, 16]. The PAQUID study is an ongoing cohort with the objective of describing the evolution of cognitive functions to identify predictive signs of and risk factors for dementia, and to study dependence in older adults, its risk factors, and consequences. This project started in 1988; 5554 individuals aged 65 years or above, living at home, and randomly selected from the electoral lists of Gironde and Dordogne, France, were invited to participate. Among them 3777 (68%) agreed; they were representative of the elderly population of these two areas. In 2013–2014, 231 PAQUID participants still alive underwent the 25-year follow-up among them 197 were invited to participate in the oral sub-study(34 participants were not invited to participate due to a delay in the start of the study; see [15, 16] for more details). Those who agreed were visited by a dentist. Previous analyses have shown that the participants of the oral sub-study (*N* = 90) were similar in terms of age and sex to those who did not participate (*N* = 117), but were less likely to be institutionalized and to have a low education level [15].

### Outcomes of interest

Nutritional status and oral health were assessed by two dentists at the participants’ living place.

Standardized questionnaires were personally administered to either the participant or to a caregiver. The oral characteristics were recorded during an oral clinical examination in accordance with the WHO’s recommendations [17] as described elsewhere [15].

Nutritional status was assessed using the full version of the MNA, which consists of a general health assessment, a dietetic assessment, anthropometric measures, self-perception of health and nutrition, and a global evaluation involving questions on living independently, prescription drug use, psychological stress, acute disease, mobility, dementia, and skin conditions [18]. The score, ranging from 0 to 30, is interpreted as follows: MNA ≥ 24 as normal nutritional status; 17 ≤ MNA ≤ 23.5 as at risk of malnutrition, and MNA < 17 as malnutrition [19].

Oral health was evaluated using the Decayed-Missing-Filled teeth index (DMFT index) on 32 teeth [17], the number of posterior occluding pairs on natural teeth or fixed prostheses except for wisdom teeth [20], the presence of oral pain (“Currently, do you have pain in your mouth”), xerostomia (“subjective feeling of dry mouth” — Xerostomia Inventory) [21], and prosthetic rehabilitation (no tooth loss or adequate dentures, inadequate dentures, or no dentures despite tooth loss).

Teeth with carious lesions or temporary fillings on the crown or root were considered decayed (D). Teeth were considered filled (F) if they had at least one permanent restoration (including crowns) without any caries on the crown or root [17]. Inadequate dentures were defined as the use of reliners or tissue conditioners, excessive wear of posterior teeth, lack of integrity, or problems with stability or retention [22]. Coronal and root conditions were assessed for each tooth individually. Tooth loss and the presence and adaptation of removable prostheses were assessed. The inter-observer reliability for the DMFT index, assessed using the intra-class correlation coefficient, was 0.99 (95% confidence interval = 0.98–1.00), corresponding to excellent reliability [23].

### Other characteristics

Sociodemographic and medical characteristics were obtained from the existing PAQUID database. Sociodemographic variables included sex, age, education level, and living conditions. Medical variables included the presence of comorbidities (diabetes mellitus, Parkinson’s disease, stroke, and myocardial infarction), number of medications used per day, psychotropic drug use, tobacco use, and dementia. At each follow-up visit every 2 or 3 years, dementia diagnosis was based on a three-step procedure: first, a neuropsychologist performed a neuropsychological evaluation at the participant’sliving place; second, participants suspected of dementia following this evaluation were examined by a neurologist or a geriatrician; and finally a definitive diagnosis of dementia, was made by a panel of independent neurologists, based on Diagnostic and Statistical Manual of Mental Disorders criteria (DSM-IV) [24]. Dependency was assessed using a hierarchical four-stage index that combines the Activities of Daily Living (ADL), the Instrumental Activities of Daily Living Scale (IADL), and mobility (assessed using the Rosow and Breslau scale). Participants were classified as having no dependency; mild dependency (dependent only in the Rosow scale); moderate dependency (dependent in the Rosow and IADL scales); and severe dependency (dependent in the ADL scale) [21].

### Statistical analysis

Nutritional status, oral health status, sociodemographic, and clinical characteristics were described as mean, standard error (SE), minimum (min), and maximum (max) values for quantitative variables, and percentages for qualitative variables.

We used univariate and multivariate logistic regressions to explore the association between nutritional status (MNA) as the dependent variable and oral health as explanatory variables (DMFT index, number of posterior occluding pairs, oral pain, xerostomia, and prosthetic rehabilitations). Odds ratios (ORs) were estimated with their 95% confidence intervals (CIs). MNA was categorized into two groups based on the global score obtained from the questionnaire: normal nutritional status (MNA ≥ 24, reference in the regression model) and nutritional status as the risk of malnutrition or malnutrition (MNA < 24). The association between MNA and oral health related variables, age, sex, education level, living alone, institutionalization, dementia, medication use, presence of comorbidities, or dependency was tested in univariate analyses. Variables associated with MNA in the univariate analysis with a conservative threshold of 25% were used in the multivariate analysis, along with age, which was forced in the multivariate model.

Participants who did not have complete data for dependent, explanatory, and adjustment variables (*N* = 3) were excluded from the analysis.

Statistical analyses were performed with a level of significance of 0.05 using SAS software (version 9.4) and R software (version 3.2.3).

## Results

### Participants’ descriptions

Among the 90 participants of the oral PAQUID sub-study, three were excluded. The mean age of the remaining 87 participants was 94.1 years (± 3.0; mi*n* = 90.6 years; max = 105.6 years). Most participants were females (74.7%), while almost three-quarters of the participants lived at home (Table 1). Twenty-four participants had dementia and 71.2% had other comorbidities. The number of medications used per day was 6.8 (± 3.0; mi*n* = 0; max = 16). According to the hierarchical disability scale, only one participant had no dependency. Only one participant was a current tobacco consumer.

**Table 1:** Participants ‘characteristics, PAQUID oral sub-study (*n* = 87), France, 2014

	Total		Normal nutritional status (*n* = 64)		Malnutrition or at risk (*n* = 23)	
Variables	N/Mean	(%)/(sd)	N/Mean	(%)/(sd)	N/Mean	(%)/(sd)
**Age** (years), *mean (sd)*	94.1	(3.0)	94.0	(3.0)	94.5	(3.0)
**Females** (vs. Males)	65	(74.7)	44	(68.8)	21	(91.3)
**Higher school level** (vs. No)	73	(83.9)	54	(84.4)	19	(82.6)
**Living alone** (vs. No),	44	(50.6)	33	(51.6)	11	(47.8)
**Institutionalized** (vs. living at home)	23	(26.4)	12	(18.8)	11	(47.8)
**Dementia** (vs. No)	24	(27.9)	17	(27.0)	7	(30.4)
**Number of medications per day**, *mean (sd)*	6.8	(3.1)	6.3	(3.0)	8.2	(2.8)
**Psychotropic intake** (vs. No)	35	(40.2)	24	(37.5)	11	(47.8)
Presence of comorbidities* (vs. No)	62	(71.2)	46	(71.9)	16	(69.6)
**Hierarchical disability scale**						
No or mild dependency	21	(24.1)	19	(29.7)	1	(8.7)
Moderate dependency	35	(40.2)	27	(42.1)	8	(34.8)
Severe dependency	31	(35.7)	18	(28.1)	13	(56.5)
**DMFT index > 19** (vs. ≤ 19), *n (%)*	76	(87.4)	57	(89.1)	19	(82.6)
**Posterior occluding pairs**, *mean (sd)*	1.5	(2.3)	1.8	(2.5)	0.6	(1.1)
**Oral pain** (vs. No), *n (%)*	6	(6.9)	5	(7.8)	1	(4.3)
**Xerostomia** (vs. No), *n (%)*	21	(24.1)	9	(14.1)	12	(52.1)
**Prosthetic rehabilitation**, *n (%)*						
No tooth loss or adequate dentures	7	(8.1)	6	(9.4)	1	(4.3)
Inadequate dentures	41	(47.1)	31	(48.4)	10	(43.5)
No dentures despite tooth loss	39	(44.8)	27	(42.2)	12	(52.2)

*DMFT* Decayed-Missing-Filled Teeth index. *Sd* Standard deviation. *MNA* Mini Nutritional Assessment

### Nutritional status

The mean MNA score was 25.5 (± 3.7). Three-quarters of the participants (*n* = 64; 73.6%) had a normal nutritional status, 22 were at risk of malnutrition (25.3%), and 1 had malnutrition (1.1%).

### Oral status

The mean DMFT score was 26.5 (± 5.3) with a mean of 3.6 decayed teeth (± 4.9), 4.8 filled teeth (± 5.3), and 18.1 missing teeth (± 9.2). The mean number of posterior occluding pairs was 1.5 (± 2.3). Oral pain and xerostomia were reported in 6.9% and 24.1% of the participants, respectively. In the maxilla, 10 participants (11.5%) had all their teeth, 51 (58.6%) had partial tooth loss, 26 (29.9%) had total tooth loss and 50 (57.5%) used dentures. In the mandible, 11 participants (12.6%) had all their teeth, 58 (66.7%) had partial tooth loss, 18 (20.7%) had total tooth loss and 42 (48.3%) used dentures. Globally, 54 participants (62.1%) used dentures but, 84.0% of the maxillary dentures and 85.7% of the mandibular dentures were inadequate. There were also a few people (*n* = 5) who did not use their dentures during meals.

### Association between oral health and nutritional status

The univariate analysis demonstrated that xerostomia was associated with being malnourished or at risk of malnutrition (OR = 6.67; 95% CI = 2.26–19.63; *p* = 0.0006) as well as a one-unit decrease in the posterior occluding pairs (OR = 0.69; 95% CI = 0.49–0.99; *p* = 0.041) (Table 2).

**Table 2:** Factors associated with the probability of malnutrition or risk of malnutrition defined by a MNA score < 24, PAQUID oral sub-study (*n* = 87), France, 2014

	Univariate analysis			Multivariate analysis		
Variables	OR	95% CI	*p*-value	OR	95% CI	*p*-value
**Age** (years), *mean (sd)*	1.06	[0.90 – 1.23]	0.485	1.10	[0.90—1.34]	0.150
**Females** (vs. Males)	4.77	[1.23 – 31.65]	**0.047**	4.55	[0.75 – 48.43]	0.326
**Higher school level** (vs. No)	0.88	[0.26 – 3.50]	0.843			
**Living alone** (vs. No),	0.86	[0.33 – 2.24]	0.759			
**Institutionalized** (vs. living at home)	3.97	[1.42 – 11.36]	**0.009**	1.38	[0.28 – 6.51]	0.535
**Dementia** (vs. No)	1.18	[0.40 – 3.31]	0.752			
**Number of medications per day**, *mean (sd)*	1.23	[1.05 – 1.48]	**0.015**	1.07	[0.86 – 1.35]	0.142
**Psychotropic intake** (vs. No)	1.53	[0.58 – 4.03]	0.388			
**Presence of comorbidities**^a^ (vs. No)	0.89	[0.32 – 2.65]	0.834			
**Hierarchical disability scale**			**0.020**			0.186
No or mild dependency	1.00	-		1.00	-	
Moderate dependency	2.81	[0.62 – 20.05]		3.77	[0.57 – 37.41]	
Severe dependency	6.86	[1.60 – 47.94]		6.83	[0.87 – 74.81]	
**DMFT index > 19** (vs. ≤ 19), *n (%)*	0.58	[0.15 – 2.21]	0.428			
**Posterior occluding pairs**, *mean (sd)*	0.69	[0.49 – 0.99]	**0.041**	0.66	[0.37 – 0.98]	0.089
**Oral pain** (vs. No), *n (%)*	0.54	[0.06 – 4.81]	0.579			
**Xerostomia** (vs. No), *n (%)*	6.67	[2.26 – 19.63]	**0.0006**	8.79	[2.38 – 39.10]	**0.002**
**Prosthetic rehabilitation**, *n (%)*			0.618			
No tooth loss or adequate dentures	1.00	-				
Inadequate dentures	1.94	[0.21 – 18.07]				
No dentures despite tooth loss	2.67	[0.29 – 24.64]				

*DMFT* Decayed-Missing-Filled Teeth index. *Sd* Standard deviation. *MNA* Mini Nutritional Assessment. *OR* Odds Ratio. *CI* Confidence Interval

^a^ Presence of diabetes, Parkinson's disease, history of myocardial infarction or stroke

Sex, posterior occluding pairs, xerostomia, living in an institution, number of medications used per day, and the disability scale were identified by the univariate analysis at the 25% threshold and were included in the multivariable model.

After adjustment for these variables and for age, only xerostomia remained associated with being malnourished or at risk of malnutrition was associated with xerostomia (OR = 8.79; 95% CI = 2.38–39.10; *p* = 0.002) (Table 2).

## Discussion

This study demonstrated that about 25% of these participants aged 90 years or over were at risk of malnutrition while only 1.1% was malnourished. Together, the prevalence was quite high compared to the prevalence reported in the literature and corresponded more to prevalence rates found in hospital settings [25]. In general settings, Torres et al. [6] reported a prevalence of poor nutritional status (at risk of malnutrition and malnutrition, assessed by MNA) of 7.4–14.8% in subjects having a mean age of 75.5 years, while Nykänen et al. [10] reported a prevalence of possible malnutrition (at risk of malnutrition and malnutrition, assessed by MNA short form) of 15% in a sample with a mean age of 81 years. This difference could be explained by the fact that the participants in the present study were older than those in these previous studies. This is indeed, to our knowledge, the first study reporting findings about such aged population.

As discussed elsewhere [15], our study sample presented with important dental care needs: 3.6 decayed teeth which is well above those of national studies conducted in other countries, with untreated caries representing a small part of the DMFT (DT ≤ 0,5), even in people > 75 years old [26]; about 85% of the prosthesis not adapted which is common, even in younger adults [22]. However, these conditions were not associated with poor nutritional status.

Xerostomia was independently associated with malnutrition or risk of malnutrition in our population. This was in concordance with the study conducted by Nykänen et al. [10] where dry mouth was independently associated with the risk of malnutrition. Xerostomia may influence nutritional status due to oral discomfort, taste aberrations, dental caries, oral infections, and difficulty retaining prostheses. A recent study found that xerostomia was reported by 24% of the participants with the mean age of 67.6 ± 6.1 years, and tended to be associated with a two-fold increased risk of incident malnutrition (adjusted hazard ratio = 1.99; 95% CI: 0.93–4.28; *p* = 0.077) [27]. In addition, a randomized study examined the effectiveness of interventions focusing on xerostomia and nutritional status among home-care clients aged 75 years or above who were malnourished or at risk of malnutrition [28]. Among the participants who received both interventions, xerostomia decreased by 30% while malnutrition or risk of malnutrition decreased by 61%. These studies, along with the present study, indicate the importance of xerostomia screening and care in older adults with nutritional problems.

A recent meta-analysis showed that poor nutritional status was associated with lower number of pairs of teeth/functional teeth units [14]. This is in contrast with our study, where a one-unit decrease in the posterior occluding pairs was associated with the nutritional status in univariate analysis but this association did not remain after adjustment. Although it is clear that chewing problems can influence dietary intake and food choices, they may not necessarily lead to a poor nutritional status. For example, fruits and vegetables avoided by edentulous people can be replaced by juices. The consumption of particular foods appears to influence quality of life more than nutritional status [29].

In our study, prosthetic rehabilitation was not associated with malnutrition. Interventional studies are discordant on this topic. For instance, a 2000 study by Moynihan et al. [25] found no dietary changes after fixed or removable prosthetic rehabilitation. In contrast, McKenna et al. [30] demonstrated a significant increase in the MNA score 12 months after denture use in a randomized trial. Further research is needed to clarify the impact of prosthetic rehabilitation on nutritional status.

The limitations of our study included a restricted sample size, due to the extremely old study population. This limitation could have resulted in a lack of power and precision reflected by our wide Cis. Any conclusions drawn from the present data should, if possible, be replicated with a larger sample size. Furthermore, it was a cross-sectional study that could not establish causality. Finally, the participants were from a cohort initiated in 1988, and less than half people from this cohort agreed to participate in the oral sub-study, so the participants were selected as a necessity. Therefore, caution must be exercised while generalizing the results of this study to the entire population.

Our study has several strengths. First, although most studies on geriatric population’s oral health were conducted in institutionalized individuals, our study was carried out mainly in participants living at home. Indeed, PAQUID initially included people living at home and at the 25-year follow up, only about one quarter of them were institutionalized. As a result, we could study the nutritional status and oral health in a population that is often neglected in studies due to difficult access. We were particularly interested in the home-based older adults because of undetected oral health problems in this population due to limited contact with the health care system. Second, we focused on a very old population aged ≥ 90 years who was never studied before. Third, data were collected by trained examiners using validated and reproducible indices. Finally, we considered potential confounding factors while investigating the association between oral health and nutritional status. Therefore, our multivariate analysis provided a robust basis for the current knowledge.

In conclusion, nutritional status and oral health are often poor in older adults. Our study highlights the importance of oral health, particularly xerostomia, associated with nutritional status, in older adults. Systematic joint screening of oral and nutritional problems in older adults could lead to improvements in their general health and quality of life. Information and training of health professionals working in institutions or who are in contact with older adults could improve professional practices, including screening and care of xerostomia. Our findings need to be supported by additional longitudinal and interventional studies, addressing both aspects, to provide a better understanding of the relationship between oral health and nutritional status, and to improve the general health and quality of life in older adults.

## Data Availability

The datasets analysed during the current study are not publicly available because French regulatory imposes controlled access to participants' personal data but are available from the corresponding author on reasonable request.

## Abbreviations

CI: Confidence interval
DMFT: Decayed, missing, filled teeth
IADL: Instrumental Activities of Daily Living Scale
MNA: Mini nutritional assessment
SD: Standard deviation
